# A proposed unified mitotic chromosome architecture

**DOI:** 10.1073/pnas.2119107119

**Published:** 2022-05-11

**Authors:** John Sedat, Angus McDonald, Herbert Kasler, Eric Verdin, Hu Cang, Muthuvel Arigovindan, Cornelis Murre, Michael Elbaum

**Affiliations:** ^a^Department of Biochemistry and Biophysics, University of California, San Francisco, CA 94158;; ^b^Micron School of Materials Science and Engineering, Boise State University, Boise, ID 83725-2090;; ^c^Buck Institute for Research on Aging, Novato, CA 94945;; ^d^Salk Institute for Biological Studies, La Jolla, CA 92037;; ^e^Department of Electrical Engineering, Indian Institute of Science, Bengluru 560012, India;; ^f^Division of Biological Sciences, University of California San Diego, La Jolla, CA 92092;; ^g^Department of Chemical and Biological Physics, Weizmann Institute of Science, 760001 Rehovot, Israel

**Keywords:** cell biology, chromosome structure, biophysics and computational structure analysis, computational structure analysis

## Abstract

A molecular architecture is proposed for a representative mitotic chromosome, human chromosome 10. This architecture is built on an interphase chromosome structure based on cryo-electron microscopy (cryo-EM) cellular tomography [J. Sedat *et al.*, *Proc. Natl. Acad. Sci. U.S.A.*, in press], thus unifying chromosome structure throughout the complete mitotic cycle. The basic organizational principle for mitotic chromosomes is specific coiling of the 11-nm nucleosome fiber into large scale, ∼200-nm interphase structures, a Slinky [https://en.wikipedia.org/wiki/Slinky; motif cited in S. Bowerman *et al.*, *eLife* 10, e65587 (2021)], then further modified with subsequent additional coiling for the final mitotic chromosome structure. The final mitotic chromosome architecture accounts for the dimensional values as well as the well-known cytological configurations. In addition, proof is experimentally provided by digital PCR technology that G1 T cell nuclei are diploid with one DNA molecule per chromosome. Many nucleosome linker DNA sequences, the promotors and enhancers, are suggestive of optimal exposure on the surfaces of the large-scale coils.

## Significance

The significance of this proposed mitotic chromosome architecture is that a specific, sequenced chromosome, human chromosome 10, can be built into a specific architecture that accounts for the dimensional values and cytological descriptions. Since this molecular architecture is an extension of the interphase chromosome structure, a coiling of the 11-nm nucleosome fiber with further coiling, a unifying molecular structure motif is present throughout the entire mitotic cycle, interphase through mitosis.

Packing the DNA of a given chromosome, with multiple centimeters of DNA for an average human chromosome (1–3), into a mitotic chromosome some microns in length requires a length compression of 10 to 20,000, a challenging problem. There are many models for mitotic chromosome architecture (reviewed in refs. 4 and 5), most emphasizing various-sized loops (6), but most models do not fully account for the length compression that is required to satisfy the packing density. In addition, most mitotic chromosome models do not account for the cytological modifications seen in the familiar chromosome banding spreads, a requirement for an acceptable mitotic chromosome architecture.

This paper proposes a mitotic chromosome architecture that fully satisfies the length compression, dimensional values, and cytological chromosome structure changes for a representative chromosome, human chromosome 10 (7). This chromosome, whose DNA is fully sequenced, has a DNA size of 46 mm that is packed into a mitotic chromosome (on average 5 to 6 μm in length), thus a length compression of about 10,000, suggesting an important boundary condition for the architecture.

The proposed mitotic chromosome architecture is built on an interphase chromosome architecture (1). In brief, a scanning transmission electron microscopy cryo-electron microscopy (cryo-EM) nucleus tomogram, preserving the interphase nuclear structures by the cryo procedures and then suitably processed by deconvolution (1, 8–10) was made. Throughout the nucleus, a 100- to 300-nm coiled nucleosome 11-nm fiber was documented. This interphase structure was termed a Slinky (see ref. 2, motif cited in ref. 3) and is described in detail in ref. 1.

**Fig. 1.**
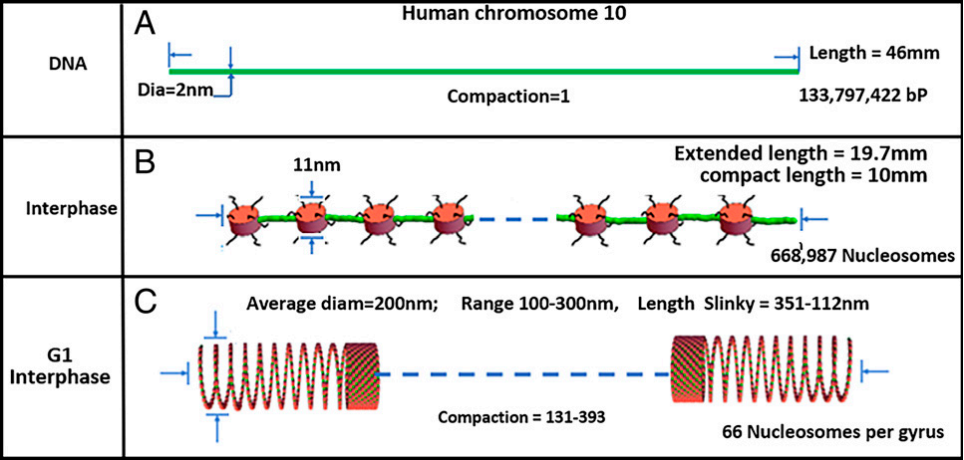
This figure documents human chromosome 10 from DNA level to Slinky nucleosome coil. (*A*) Depicts human chromosome 10 DNA, properly scaled. (*B*) Shows the nucleosomes (red disks, scaled) with the green linker DNA extended while it is possible the nucleosome chain can be compressed with the linker DNA more compactly as green hairpins. The histone tails are black wavy lines. Blue dash denotes continuation of the structure. (*C*) Shows human chromosome 10 nucleosome coiling to form a Slinky with variable diameter, compaction, and Slinky length. The extended nucleosome length is 669k nucleosomes (11 nm [the nucleosome size]) + 669k nucleosomes (18.4 nm [linker DNA size]), while compact nucleosome fiber is 669k nucleosomes (11 nm) + 669k nucleosomes (2 × 2 nm [DNA size for linker]). The number of Slinky and race track Slinky gyri can be calculated assuming 669k nucleosomes/66 nucleosomes/gyrus = 10,136 gyri. The compaction is Slinky length/DNA length, where the Slinky length is 10 mm [the compressed nucleosome fiber length]/Slinky gyrus circumference [to give the number of Slinky gyri] × 11 nm [nucleosome size; the gyrus size assuming maximal Slinky packing].

Fig. 1 summarizes the interphase chromosome structure. First, human chromosome 10 DNA is depicted in Fig. 1*A*). Second, most DNA in the nucleus is organized into the familiar nucleosomes, with extended linkers shown in Fig. 1*B* or with linkers compressed as hairpins to compact the nucleosome 11-nm fiber. The nucleosome can be rotated so the 11-nm face is oriented in the fiber direction (see Fig. 3 *A* or *C* as an example), for space considerations. There are 669,000 nucleosomes in human chromosome 10. Third, the nucleosome fiber is further coiled into 100- to 300-nm structures, defined as Slinky, with 200 nm the average diameter. The human chromosome 10 compacted by the Slinky is ∼112 to 351 μm in length as shown in Fig. 1*C*. The Slinky is coiled more tightly to form heterochromatin or pulled out variably for transcription.

This paper uses a package of software, described in *Materials and Methods*, to sequentially coil multiple helices for quantitative modeling as a tool to explore mitotic chromosome architecture. This tool was also used in the interphase chromosome structure studies (1). The software models to an accurate (molecular) scale. Once mitotic chromosomes are built, they can be analyzed in any dimension or size scale.

The classical picture of the human genome (as a general example) is one of diploid organization and one DNA molecule per sister chromatid (12, 13). Nevertheless, there are still a few questions related to this generality (14). To clarify this boundary condition, essential for the proposed mitotic chromosome structure, a careful experimental protocol using modern digital PCR technology with G1 T cells was made, giving rise to a clear answer. The results show T cells (in G1) are strictly diploid, with one DNA molecule per single sister chromatid, the classical result.

## Results

### Direct Experimental Evidence That a Single Sister Mitotic Chromosome Has a Single DNA Molecule.

**Fig. 2.**
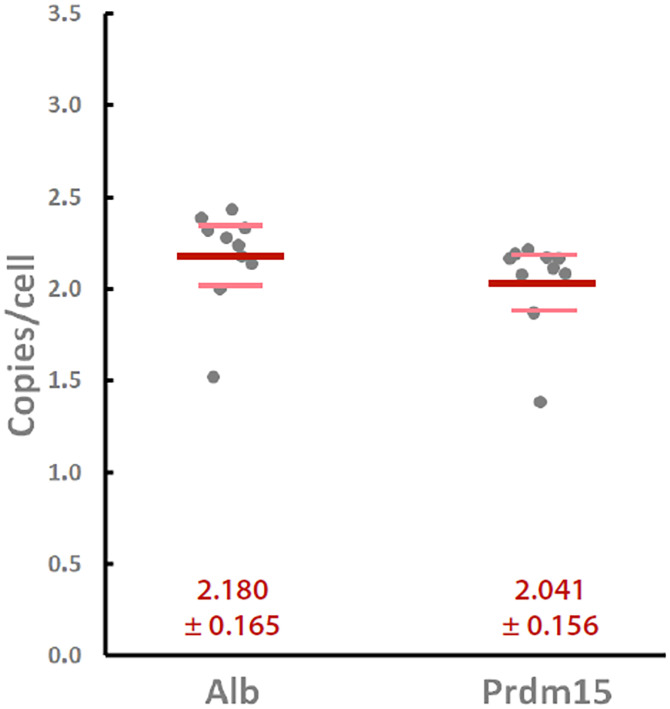
Copy number per cell (dark gray symbols) of two different genes that appeared once per haploid genome, Albumin (Alb; *Left*) and Prdm15 (*Right*), as assessed by droplet digital PCR in murine CD4^+^CD8^+^ thymocytes. Ten separate samples of 1 million CD4^+^/CD8^+^/CD3^lo^/CD5^lo^ thymocytes were sorted from two different C57BL/6 mice, and their genomic DNA was prepared with radioisotope-based recovery tracking as described in *Materials and Methods*. A precisely known number of input cell equivalents was then amplified using primers and TaqMan probes specific for the indicated genes, and copies per cell were calculated based on the counts of positive droplets. Bold red and lighter pink lines represent the means and 95% confidence intervals of the measurements, respectively.

We directly experimentally tested the classical tenet that a single mitotic sister chromosome has only one copy of that chromosome’s DNA, or is uninemic (14). A stochiometric experiment was carried out. In brief, as described in *Materials and Methods*, multiple samples of T cells in G1/G0 were cell sorted. The T cells were from a highly inbred mouse line that is homozygous for all genetic loci and, as such, had homologous chromosomes essentially with the same DNA sequence. The samples were spiked with a radioactive fragment of λ DNA to control for losses, and the DNA was extracted, making sure that the proteins did not mask DNA sequences. Carefully selected single-copy genetic loci were used for digital PCR analysis. Fig. 2 shows the results indicating that cells in G1/G0 have one copy of a DNA molecule per chromosome, a classical tenet in biology, and a boundary for the mitotic chromosome structure.

### Slinky Modification Allows Further Interphase Chromosome Compaction: Race Track Slinky.

We tried to use the interphase chromosome Slinky structure that had been described (1) to model, by coiling this structure into a dimensioned human chromosome 10 mitotic chromosome. We used the coiling software described in *Materials and Methods*. The answer quickly showed that the compaction was off by an order of magnitude—only compacted 1,000 times—a common problem for mitotic chromosome architectures.

**Fig. 3.**
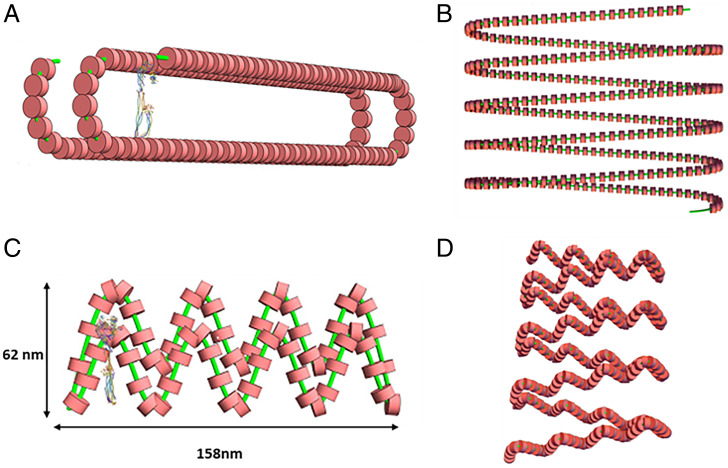
The race track Slinky coil is a modified Slinky helix of the nucleosome fiber. (*A*) Depicts a single race track Slinky gyrus accurately to scale, with a Condensin I (11) for scale. (*B*) Shows a race track Slinky helical coiled region with coils more compressed. The minor axis of the race track is 50 nm, with the major axis about 285 nm. (*C*) Shows a folded race track Slinky with short axis and long axis dimensioned. (*D*) Shows a folded race track Slinky as a short coiled structure. The green lines are the chromosomes and the red structures are the nucleosomes.

Study of the Slinky cross-section showed that its hollow structure, on average 200 nm in diameter, was cylindrical and could be modified so its shape was indented while still preserving the circumference, as shown in Fig. 3*A*. We noted that Slinky could vary its diameter and nucleosome packing (see Fig. 1*B*) as well as rotation of the nucleosome (see Fig. 3 as examples), and we choose 66 nucleosomes/gyrus as a reasonable packing for subsequent structure changes. We define this Slinky coil as the race track Slinky to reflect its modification. The cross-section of the race track Slinky can be variably compressed, reducing its short axis but lengthening its long axis. The compressed Slinky is oval, shaped like a race track; the ends have a circular geometry while the sides are straight lines, resulting in a short axis of about 50 nm and a long axis of ∼290 nm. A race track Slinky single gyrus is shown in Fig. 3*A* (and legend), with an extended race track Slinky coil in Fig. 3*B*. The elongated cross-section could be (possibly) further compacted by helically twisting the structure shown in Fig. 3 (in essence a fat ribbon) so that it is a now very dense (reduced in cross-sectional size) rope—packed into a roughly circular, dense structure. This aspect of the structure was not further modeled into the mitotic chromosome. The race track as shown in Fig. 3*A* was incorporated into the modeling software, as described in *Materials and Methods*, for the race track Slinky architecture.

An ellipse was also considered for the compression of Slinky into a modified Slinky. A problem with the Slinky ellipse is the tight turns at the top of the ellipse; the nucleosomes are differentially compressed. Some of the compression can be taken up by the flexible extendable nucleosome linker DNA, but a more interesting modification is to reduce the compression by rotation of the nucleosome by 90° so the nucleosome is now a 5-nm disk wedge (instead of a 11-nm disk) in the plane of the nucleosome fiber, better accommodating the tight turn. The modeling software did not have this flexibility. The ellipse also had a reduced interior volume, restricting protein occupation. The ellipse was abandoned for the modification of the Slinky structure.

The race track Slinky was further considered for additional structures, primarily further coiling of the race track Slinky. However, further coiling showed that the centers of such coiling were very dense, so modifications of the race track Slinky were necessary. Fig. 3*C* shows that additional folds could be indented into a folded race track Slinky—in essence creating loops, further compressing the long axis of the race track; the folded race track Slinky coil of such structure is shown in Fig. 3*D*. As shown below, such a structure now allowed a mitotic chromosome to be built.

### The Folded Race Track Slinky Structure Can Be Further Coiled to Form the Mitotic Chromosome.

**Fig. 4.**
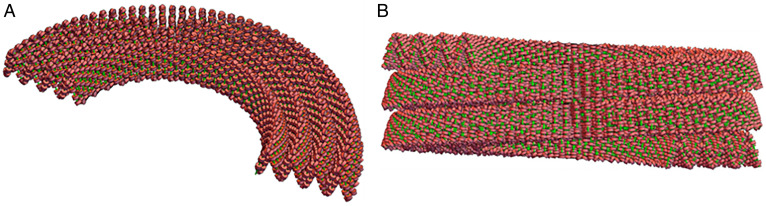
Folded race track Slinky coils can be further helically coiled to form a final mitotic coil helix, the final coil of the mitotic chromosome. (*A*) One-half helical turn of the final mitotic coil using the folded race track Slinky, drawn to scale, is shown. The width of the folded race track Slinky coil (the minor axis of the folded race track) is ∼60 nm, and the final mitotic coil of coils is the width of the mitotic chromosome, ∼0.6 μm. (*B*) Shows two and a half helical turns of final mitotic coil. Nucleosomes of the folded race track Slinky coils are visible. The green lines are the chromosomes and the red structures are the nucleosomes.

The folded race track Slinky structure depicted in Fig. 3*D* can be further helically coiled about the long axis of the folded race track Slinky to additionally compact the structure into a mitotic chromosome. This new additional coiling is shown in Fig. 4 as a half-turn of the coil (Fig. 4*A*) and as two and a half turns (gyri) of the helical twists (Fig. 4*B*). These are properly dimensioned, and the nucleosomes of the previous coils are visible. We define this additional coiling as the final mitotic coil.

**Fig. 5.**
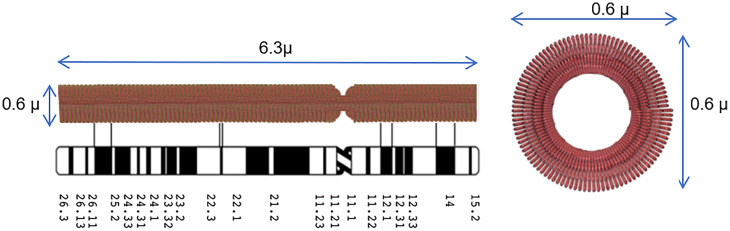
Human chromosome 10 mitotic chromosome structure. *Left*: Shows 97 final mitotic coil helix coils that build the human chromosome 10 mitotic chromosome for a length of about 6 μm and a diameter of about 0.6 μm. The centromere constriction separates the two unequal arms (scaled) of human chromosome 10, a metacentric mitotic chromosome. *Lower*: Depicts the banding cytology map for human chromosome 10 (7), as accurately aligned with the structure as possible. See (7, 15) for spread mitotic chromosome structures. *Right*: A cross-section of human chromosome 10 mitotic chromosome, properly dimensioned.

It is now possible to build with the final mitotic coil structure into the human mitotic chromosome 10 as shown in Fig. 5*A*. In order to accommodate the 669,000 nucleosomes in human chromosome10, there are ∼97 (96.5) full helical turns of the final mitotic coil to be made, resulting in a chromosome length of about 6 μm by ∼0.6 (0.59) μm in diameter. This length/diameter of the final mitotic coil nucleosome packing satisfies the required length compression, while the length/diameter matches the observed approximate cytological chromosome spread lengths/widths (see ref. 7). Fig. 5*B* shows the cross-section of the mitotic chromosome with its hollow center [see the fourth point in the *Discussion* for comparison of current mitotic chromosome experimental data; cf (15) and references therein on this point]. We note the very densely packed nucleosomes. Thus, a credible mitotic chromosome architecture results.

## Discussion

There are several points to bring out in a discussion of the proposed mitotic chromosome architecture.

First, there is a link of the architecture to the well-known mitotic chromosome cytology spreads (see ref. 7), the chromosome manipulation procedures to compact some regions while decondensing other regions that stain differentially in a reproducible manner. Genetic locations, transcription events as well as deletions, while functioning at the DNA level, are localized on the mitotic chromosome bands (7). The banding patterns—broad as well as thin bands—for human chromosome 10 are shown in Fig. 5. What is seen is that there is a straight boundary line clear across the mitotic chromosome width, delineating the stained band from the decondensed mitotic chromosome regions. We conjecture that the bands reflect the underlying coils of the final mitotic coil; for example, the thin bands are one turn (gyrus) (about the right size), while a broad band is an integral number of gyri of the final mitotic coil. Sister chromatid exchange, a recombination phenomenon (16), is another example of cytology and architecture linkup. Sister chromosome exchange procedures differentially stain parental chromatids so that mitotic recombination exchanges are readily visualized (17). The exchanges show dark bands reciprocally moved to light sister chromatids, but the boundary of the dark/light exchange bands cuts straight across the mitotic chromosome width, like the above cytological banding patterns (see ref. 16). The sharp dark/light lines, we conjecture, are the boundaries of the final mitotic coil gyri. All these features of our mitotic chromosome model reflect an essential feature of mitotic chromosome architecture: its colinearity with its DNA.

**Fig. 6.**
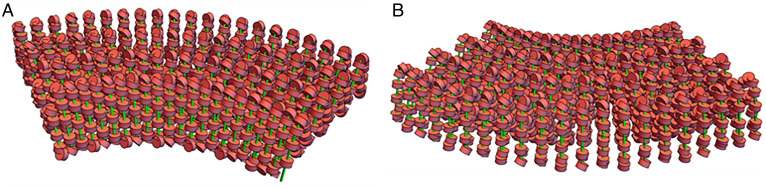
Faces of the final mitotic coil winds highlight optimally exposed nucleosome linker DNA. (*A*) Shows the enlarged inside surface of the final mitotic coil half-coil. See Fig. 4 for orientation. The enlarged back face of the final mitotic coil is shown in (*B*). The green lines are the chromosomes and the red structures are the nucleosomes.

Second, the mitotic chromosome architecture possibly positions transcriptional events optimally for the next G1. The faces of the final mitotic coil gyri have densely packed nucleosome linker DNA sequences, the promotors and enhancers sequences, as shown in Fig. 6. The mitotic chromosome cytology banding patterns described above suggest that many related genetic pathways are located on specific bands (7). We propose that the faces of the final mitotic coil gyri would expose the nucleosome regions in a more easily searched fashion (in early G1) to optimize the transcription process sequence.

Third, we built the mitotic chromosome architecture in a specific, almost rigid crystalline appearance; this appearance reflects the necessity of showing the mitotic chromosome/structure with all the 670k nucleosomes. The reality is that there is likely a great deal of architectural flexibility. First, the nucleosomes shown in Fig. 1*B* have linkers extended (some even with nucleosome-free DNA regions), which would extend the nucleosome fiber; alternatively, the nucleosomes could be compressed with hairpin linkers. Second, different numbers of nucleosomes could be coiled in the Slinky, changing the Slinky diameter, a fact that was observed in the experimental data (1). Then, the race track Slinky could be modified by changes in the major/minor axes of the race track, the extreme of which is shown in Fig. 3 *C* and *D*. This would change the packing density. Third, the final mitotic coil could be coiled more tightly or loosely to change the mitotic chromosome structure. Finally, pulling out the folded race track Slinky and the final mitotic coil in various places (changing the helical pitch even for short distances) also changes the mitotic chromosome structure. All coiling modifications could be working in concert in various combinations. The resulting mitotic chromosome structure would have many bulges/bumps along its length. The many chromosomal proteins (18) are likely distributed differentially along the length of the mitotic chromosome, and they might have a differential packing effect on the mitotic chromosome (see refs. 7 and 18), further modifying the structure. We make no statement as to handedness; essentially, all these levels could have a specific left- or right-hand coil, and that hand could, in principle, flip, though the transition region would be locally disorganized.

We are stunned by the density of the nucleosome packing that is required to satisfy the boundary conditions. To increase the packing density, every packing feature has to be used to get the structure to fit the mitotic chromosome, length compression, and sizes. There is little room for slop.

We are at the upper end of the dimensions of human mitotic chromosome 10, and reduction of the dimensions may be possible. There is flexibility in the structures for accommodation in the tight packing density. In the tight turns (Fig. 3*C*) and inside the mitotic chromosome hole (Figs. 4 and 6*A*), for example, the substantial flexible linker DNA on each side of the nucleosome will allow optimal movement. Flipping the nucleosome by 90° (now 5.5 nm in size) at the ends of the short axis of the modified race track will allow for a tighter final mitotic coil coiling and reduction of mitotic chromosome diameter and length. The pitch of the final mitotic coil or the number of the race track Slinky gyri/final mitotic coil gyrus will change the packing density/mitotic chromosome size also.

It is formally possible that some (or many) nucleosomes are removed from the Slinky or the race track Slinky structures in the compacting stages of the mitotic chromosome so that bare DNA can pack more densely. No evidence exists for this possibility. We are careful to state that other mitotic chromosome architecture schemes are possible but are not explored.

Fourth, while we are careful to not say how such coiling is molecularly specified for this mitotic chromosome architecture, such coiling is used in many places in cell biology, such as in the structure of Myosins (12). The abundant mitotic chromosome proteins (18) are likely to make such coiling possible.

The major chromosomal proteins, the Condensins I and II (15, 19–23), can be brought into the architecture, and the modified race track Slinky structure lends itself for this protein interaction. The latest Condensin I structure from the Protein Data Bank (21) has a major axis of 34 to 36 nm, which comes close to matching the race track Slinky short axis of about 50 nm as seen in Fig. 4 *A* and *B* (drawn to scale). Condensins I and II, similar in structure, are shaped to interact with, bind, and pull together the sides of the short axis of the race track (as discussed in refs. 15 and 19–23). A stick figure model of Condensin taken from (22) can also dock in the race track (Fig. 4*A*) for comparison. Cohesin, a very similarly shaped molecule (11, 24), can also interact with the race track. A publication describes a careful study of the stoichiometry of Condensin I and II throughout the cell cycle (15). Indeed, the large hole in the center of the final mitotic coil of the mitotic chromosome largely matches the data of (15). This reference showed strong evidence for the bulk of the DNA in the periphery of the mitotic chromosome and the Condensins in the center, resulting in essentially a hollow structure. There are other places for key proteins to bind and aid in the structure; Histone H1 (25, 26) could bind two nucleosome pairs, above and below—in trans—the multiple folds in the folded race track Slinky (Fig. 3*C*), which could consolidate/strengthen this structure. In summary, the mitotic chromosome structure suggests that a protein scaffold would be an essential component of this architecture.

Fifth, it is formally possible that several (two to four) of the final mitotic coil gyri associate, in phase, through protein associations to form larger structures, possibly of the order of 0.2 to 0.3 μm (see Fig. 3). These larger structures have been seen in some mitotic chromosome preparations (27), including many in the Sedat laboratory (unpublished) over the years.

Sixth, the very dense packing that is required to compress the nucleosomes into a mitotic chromosome suggests that biochemical perturbations such as fixation or chromosome isolation buffers (see ref. 5) are a major problem. This very high density of packing will facilitate crosslinking, bringing structures artificially together while pulling other structures apart with a distorted final structure. The solution will likely be structure-preserving cryo-EM tomography.

Seventh, the proposed mitotic chromosome architecture, with its dense, closely packed helices, emphasizes that rigorous structure biology interpretation tools will be required to adequately solve the structure with proof. One can easily get lost among the multiple coils, especially with reduced resolution or inadequate Z resolution, leading to a false final structure. For an example, it is difficult, even with stereo viewing, to convince oneself that the final mitotic coiling of the mitotic chromosome is not a two-stranded structure, though we know by building the structure that this cannot be true. The final mitotic chromosome structure awaits study by cryo-EM tomography.

### Major Conclusion.

**Fig. 7.**
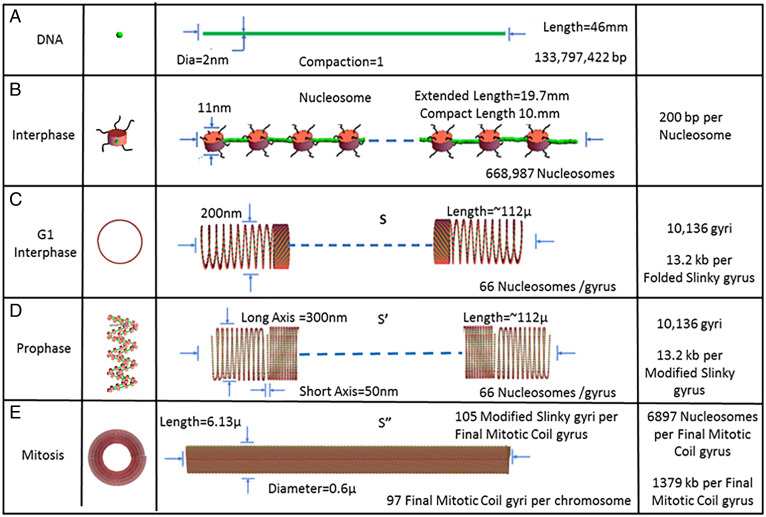
A summary of the unified architecture changes, using the helical multiple coiling motif taking place throughout the cell cycle, interphase through mitosis, a complete cell cycle for human chromosome 10. (*A*) Shows the DNA level, and (*B*) shows the nucleosome 11-nm organization with extended linker DNA (green). (*C*) Depicts the Slinky level with the coiled nucleosomes tightly packed with 66 nucleosomes/gyrus, while (*D*) depicts indents/folds of the Slinky structure to a folded race track. (*E*) Shows the final level of organization, a mitotic chromosome based on the final mitotic coiling. The structures are drawn to scale. See Fig. 1 legend. The 6,897 nucleosomes/final mitotic coil gyrus is given by 668,987 nucleosomes/97 final mitotic coil gyri. The green lines are the chromosomes; the black squiggly lines are the histones tails; and the red structures are the nucleosomes.

Finally, in summary, we emphasize that a monolithic helical coiling architecture is used throughout the entire mitotic cell cycle from interphase through mitosis. Fig. 7 outlines a summary of the architecture as a function of the cell cycle. Fig. 7*A* shows the base level, the human chromosome 10 DNA starting point. Fig. 7*B* shows the interphase G1 next level, the extended nucleosomes. Fig. 7*C* shows the interphase G1 nucleosomes, organized into a Slinky with a 100- to 300-nm diameter, either pulled out for transcription or compacted for heterochromatin. In Fig. 7*D*, the prophase cell cycle level is shown with the Slinky indented to form a folded race track Slinky, thus further compressing the nucleosomes for additional packing. Finally, Fig. 7*E* shows the mitotic cell cycle level with the mitotic chromosome coiled into the final mitotic coil helix. This architecture satisfies the packing boundary conditions, for example, length compression and dimensions, that we outlined in the introduction. In addition, we can conclude with some certainty that one DNA molecule forms one sister mitotic chromosome and also confirms this well-known fact. Thus, there is a unity, as outlined in this proposal, for chromosomal structure—with one monolithic coiling architecture—throughout the entire cell cycle.

## Materials and Methods

### G1 Copy Number Determination.

Suspensions of total thymocytes at 2 × 10^7^/mL in phosphate-buffered saline (PBS)/2% fetal calf serum were prepared from two different male 8-wk-old C57bl/6 mice by crushing the organs, straining through a 40-μm nylon mesh, and Ack lysis of erythrocytes. The cells were then stained with antibodies (eBioscience) to mouse CD4 (clone GK1.4, PE), CD8 (clone 53–6.7, PE-Cy7), CD3 (clone 2C11, APC-AF780), and CD5 (clone 53–7.3, APC) at 1 µg/mL each for 20 min on ice. After washing and resuspension in PBS/2% fetal bovine serum, single preselection thymocytes (CD4^+^/CD8^+^/CD5^lo^/CD3^lo^) were sorted into preweighed (0.1 mg precision) 15-mL conical tubes at exactly 1 million cells/tube, using a BD FACSAria II cell sorter. The tubes were weighed again after sorting, and then a ^32^P-labeled bacteriophage λ-based recovery tracking probe was added to the cells, with the exact number of counts added (∼11,000 to 12,000 counts per minute [cpm], ∼50 µL) determined by the weight of the added probe solution.

The recovery probe was prepared as follows: A standard 100-µL PCR was prepared containing 10 ng bacteriophage λ DNA (NEB), 5 units of Taq polymerase (NEB), and 5 pmoles each of the following primers: TACGAACGCCATCGACTTACGCGTGCGC (5′ end) and GCCATTGCTCAGGTCGAAGAGATGCAGG (3′ end), which amplify a 6,086-bp segment of the lambda genome. The crude PCR product was gel purified from 1% agarose by spin column (Qiagen), and 200 ng of the purified amplicon was end-labeled using 10 µCi γ-^32^P adenosine triphosphate (Amersham) and T4 polynucleotide kinase (NEB). The labeling reaction was then desalted on a G-25 spin column and further precipitated with 70% ethanol, after addition of 20 µg glycogen carrier (Roche) to remove any unincorporated label. The probe was resuspended in TE-buffer at 249 cpm/mg solution at the time of addition, and the subsequent recovery determinations were adjusted for the decay of a reference sample.

After probe addition, the sorted cells (∼2 mL volume) were adjusted to 1% sodium dodecyl sulfate, 20 mM Na^+^–ethylenediaminetetraacetic acid, and 40 U/mL proteinase K (Roche) and incubated overnight at 55 °C. The digested lysate was then incubated at 80 °C for 45 min to inactivate the proteinase K and then precipitated with 70% ethanol, air dried, and resuspended in ∼200 µL TE-buffer. The tubes were weighed again to determine the mass of the resuspended DNA solution, then an ∼20-µL sample from this was weighed and counted, based on which the % recovery of each sample after digestion and precipitation was determined.

Based on this determination, 2,928 to 4,231 cell equivalents per droplet generation reaction (0.81 µL/sample, in triplicate) were assayed using the Bio-Rad QX100 digital PCR system for the copy number of two different genes, Alb and Prdm15, each of which occurs only once in the mouse genome. Sufficient droplets were acquired for each reaction to give a 95% confidence interval of less than ±5% for template concentrations according to the Poisson distribution.

Primers for the droplet digital PCR analysis were as follows: Alb fwd: GTTACCAAGTGCTGTAGTGGAT, Alb rev: GTGCAGATATCAGAGTGGAAGG, Alb probe: ACTGTCAGAGCAGAGAAGCATGGC, amplicon length = 128; Prdm15 fwd: ATGGATGTGGTCCCTGAGTA, Prdm15 rev: CCTGTCGGAGCAACATGAA, Prdm15 probe: CGCAGGTGTACTTCTTGTCACCGT, amplicon length = 113.

### Computer Modeling Software.

The computer modeling of the mitotic chromosome at all levels utilized a software package written by a Turkish engineering group (28) that allows sequential helical coiling of defined-size structures. This package requires a large workstation and Mathematica 12.3 (29). Mathematica was run on a Linux system with an Intel Core i7 CPU 2.80-GHz processor with four cores and on a Windows 7 system with an Intel Core i5 CPU 3.20-GHz processor with four cores. Output from both systems was displayed on a Samsung C27F591 monitor and NVIDIA Quadro K1200 video card and 8 GB of memory. The software was modified to take into account the race track Slinky configuration modifications. The various scripts for the software will be supplied by the authors upon request.

### Computer Display Software.

Once structures are built, they are displayed in various dimensions, with the generalized display and quantitation software package written over the years by the Agard/Sedat groups [(30); unpublished extensions by Eric Brandlund]. This software, Priism, and its extensive Help files are available through the Agard and Sedat University of California San Francisco e-mails. The display scripts are also available from the authors.

## Data Availability

Model software data are available upon request from the corresponding author.
